# Cell-free H-cluster Synthesis and [FeFe] Hydrogenase Activation: All Five CO and CN^−^ Ligands Derive from Tyrosine

**DOI:** 10.1371/journal.pone.0020346

**Published:** 2011-05-31

**Authors:** Jon M. Kuchenreuther, Simon J. George, Celestine S. Grady-Smith, Stephen P. Cramer, James R. Swartz

**Affiliations:** 1 Department of Chemical Engineering, Stanford University, Stanford, California, United States of America; 2 Physical Biosciences Division, Lawrence Berkeley National Laboratory, Berkeley, California, United States of America; 3 Department of Applied Science, University of California Davis, Davis, California, United States of America; 4 Department of Bioengineering, Stanford University, Stanford, California, United States of America; University of Wales Bangor, United Kingdom

## Abstract

[FeFe] hydrogenases are promising catalysts for producing hydrogen as a sustainable fuel and chemical feedstock, and they also serve as paradigms for biomimetic hydrogen-evolving compounds. Hydrogen formation is catalyzed by the H-cluster, a unique iron-based cofactor requiring three carbon monoxide (CO) and two cyanide (CN^−^) ligands as well as a dithiolate bridge. Three accessory proteins (HydE, HydF, and HydG) are presumably responsible for assembling and installing the H-cluster, yet their precise roles and the biosynthetic pathway have yet to be fully defined. In this report, we describe effective cell-free methods for investigating H-cluster synthesis and [FeFe] hydrogenase activation. Combining isotopic labeling with FTIR spectroscopy, we conclusively show that each of the CO and CN^−^ ligands derive respectively from the carboxylate and amino substituents of tyrosine. Such *in vitro* systems with reconstituted pathways comprise a versatile approach for studying biosynthetic mechanisms, and this work marks a significant step towards an understanding of both the protein-protein interactions and complex reactions required for H-cluster assembly and hydrogenase maturation.

## Introduction

Hydrogenase enzymes are efficient biocatalysts for the most fundamental of chemical reactions, the reversible combination of protons and electrons to form molecular hydrogen (2H^+^+2e^−^ 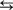 H_2_). With catalytic rates comparable to those of expensive platinum catalysts [1], hydrogenases hold great promise for use in fuel cells [2], for photosynthetic H_2_ evolution [3], for H_2_ production from carbohydrates [4], and as paradigms for synthetic catalysts [5]. They are also important for energy exchange in many ecological systems [6] and were probably key enzymes in the development of primordial biology [7].

**Figure 1:**
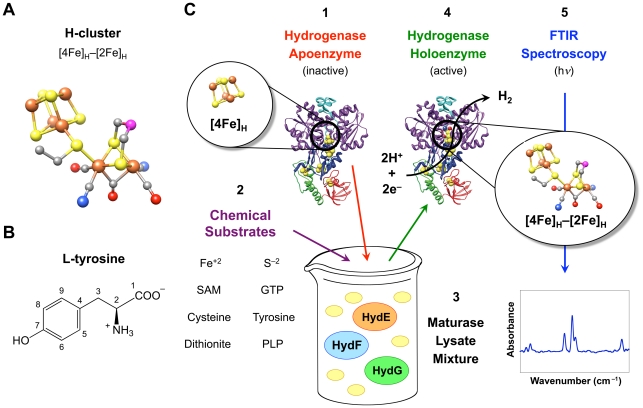
*In vitro* [FeFe] hydrogenase activation for FTIR spectroscopic analysis. (*Fig. 1A*) A ball and stick representation of the hydrogenase H-cluster. The catalytic [2Fe]_H_ cluster is joined to the cubane [4Fe]_H_ cluster, colored with the following scheme: brown (Fe), yellow (S), gray (C), red (O), blue (N), and magenta (unknown). (*Fig. 1B*) The chemical structure for L-tyrosine, with carbon atoms numbered 1–9. (*Fig. 1C*) The *in vitro* hydrogenase maturation process. For cell-free H-cluster synthesis, (**1**) CpI apoenzyme (PDB ID 3C8Y) as well as (**2**) exogenous substrates are added to (**3**) a mixture of three lysates containing *E. coli* proteins (yellow ovals) and individually produced maturases. HydE, HydF, and HydG are expressed separately to avoid H-cluster synthesis during *in vivo* maturase expression. Following hydrogenase maturation, (**4**) the CpI holoenzyme is re-purified, and (**5**) the active hydrogenase is examined using FTIR spectroscopy.

Hydrogenases contain complex [FeFe]-, [NiFe]-, or [Fe]-based catalytic cofactors that are stabilized by multiple non-protein ligands [8]. [FeFe] hydrogenases are the fastest H_2_ producers and require the H-cluster, a catalytic cofactor comprised of two iron-based clusters connected via a cysteinyl sulfur atom (Fig. 1). The cubane Fe–S cluster ([4Fe]_H_) presumably delivers electrons to the catalytic 2Fe unit ([2Fe]_H_), which contains three carbon monoxide (CO) and two cyanide (CN^−^) adducts as well as a dithiol bridging group of disputed composition [9], [10]. Three proteins called the HydE, HydF, and HydG maturases participate in the synthesis of the H-cluster and the activation of [FeFe] hydrogenases [11]. The final maturation step presumably occurs when the HydF maturase transfers the [2Fe]_H_ cluster to the hydrogenase [12], [13], likely through a positively charged channel as proposed by Mulder *et al.* [14].

One of the most intriguing mysteries has been the origin of the H-cluster CO and CN^−^ ligands, both of which are highly reactive toxins in their free states. Glycine was first considered as a plausible substrate [15], although recent and informative studies on HydG-catalyzed radical chemistry indicated that CO and CN^−^ could be generated from tyrosine [16], [17], [18]. These studies, however, were by no means definitive in showing that each of the five CO and CN^−^ ligands derive from tyrosine. The coordination of CO and CN^−^ to a hydrogenase-bound or a maturase-bound metal cluster was not demonstrated (i.e. formation of the H-cluster or a precursor thereof), and an active [FeFe] hydrogenase was not produced. Rather, the CO and CN^−^ molecules were independently detected using separate non-physiological assays. In the work by Driesener *et al.*, 20% perchloric acid was used to denature HydG and release protein-bound products, and CN^−^ was subsequently identified by derivatization methods [16]. In the work by Shepard *et al.*, CO production was detected by measuring carboxyhemoglobin, although the detectable quantities (10 µM Hb–CO) were substantially lower than the measured CN^−^ quantities (200 µM CN^−^) from reaction mixtures with similar HydG concentrations (60–65 µM) [16], [18]. While the findings in these previous studies suggest tyrosine as the source of the H-cluster CO and CN^−^ ligands [16], [17], [18], the required methods and nature of the results highlight the need for approaches in which the complete H-cluster biosynthetic pathway is reconstructed. Such methods would provide more flexibility in experimental design and enable detailed analyses of active [FeFe] hydrogenases.

The *in vitro* reconstitution of pathways for activating complex biological catalysts has historically been crucial for gaining insights into the underlying biochemistry [19]. For example, a detailed understanding of the nitrogenase accessory proteins and the synthesis of the iron-molybdenum cofactor (FeMo-co) only came after the development of cell-free approaches for nitrogenase activation [20], [21], [22]. Enabled by the discovery of the HydE, HydF, and HydG maturases [11], we previously reported the first example of *in vitro* [FeFe] hydrogenase maturation methods that could be used to examine the required substrates [23]. Although suggested substrates such as carbamoyl phosphate and glycine had no observable effects [15], [24], *S*-adenosyl methionine (SAM), cysteine, and tyrosine were essential for hydrogenase activation [23]. In our previous study, however, the maturases had been co-expressed in *E. coli*. This can lead to the *in vivo* synthesis of H-cluster precursors that associate with the HydF maturase [12], [13], [25], thereby complicating *in vitro* investigations.

In this work, we improved our previous *in vitro* system by employing separately produced maturases. Hydrogenase maturation is thus entirely dependent on the cell-free synthesis of the H-cluster. We demonstrate the utility of such methods by using tyrosine either fully or selectively labeled with ^13^C and ^15^N to generate milligram quantities of active and isotopically labeled [FeFe] hydrogenases, which are subsequently examined using Fourier Transform Infrared (FTIR) spectroscopy. In doing so, we prove that each of the H-cluster CO and CN^−^ ligands are synthesized from the carboxylate and amino substituents of tyrosine.

## Results and Discussion

Our new *in vitro* system includes inactive *Clostridium pasteurianum* [FeFe] hydrogenase (CpI) apoenzyme combined with three *Escherichia coli* cell lysates, each containing one of the maturases native to *Shewanella oneidensis* (Fig. 1). SAM, cysteine, tyrosine, ferrous ammonium sulfate (Fe^+2^), sodium sulfide (S^−2^), dithiothreitol (DTT), guanosine-5′-triphosphate (GTP), pyridoxal-5′-phosphate (PLP), and sodium dithionite are added to this mixture of proteins to reconstitute the pathway for H-cluster synthesis and hydrogenase activation.

**Figure 2:**
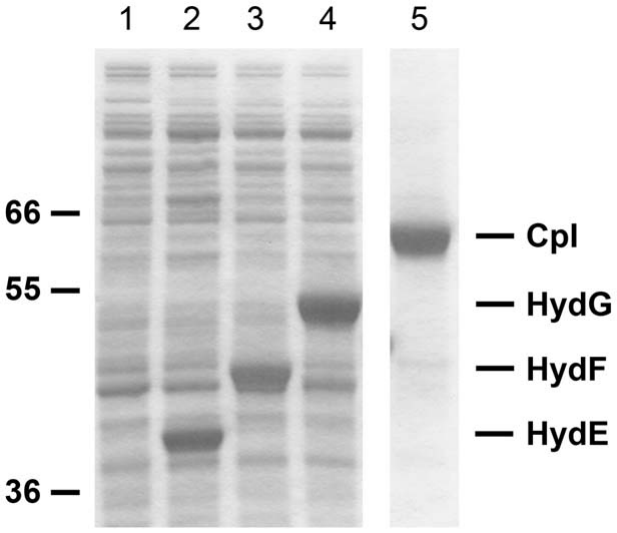
SDS-PAGE and Coomassie staining of purified CpI apoenzyme and *E. coli* lysates with heterologous maturases. All proteins were identified using the Mark12™ protein ladder (Invitrogen), and the 36, 55, and 66 kD protein standards are indicated. The control lysate from *E. coli* strain BL21(DE3) *ΔiscR* (lane 1) has no proteins produced from recombinant DNA plasmids. Maturase lysates with soluble HydE (40 kD), HydF (45 kD), or HydG (54 kD) are shown in lanes 2–4, respectively. We estimated that the cell lysates (0.25 µL of lysate loaded per lane) contained 3–15 mg·mL^−1^ of each maturase, and approximately 2.5 µg of CpI–*Strep*-tag II apoenzyme (64 kD) is shown in lane 5.

The work in this report would not have been possible without scalable methods for making large quantities of active [FeFe] hydrogenases in a cell-free environment. We recently improved the *in vivo* expression of active hydrogenases in *E. coli* [26], and we extended those methods for high-yield expression of the individual maturases and CpI apoenzyme. The maturase lysates used for *in vitro* hydrogenase maturation (Fig. 1) therefore contained high concentrations of HydE, HydF, or HydG, which we estimated to be 3–15 mg·mL^−1^ (Fig. 2). This was crucial to achieve nearly complete activation of the CpI hydrogenase (Table 1) at concentrations of ∼200 mg·L^−1^, more than 300-fold higher than with methods that lack *in vitro* H-cluster synthesis [12], [27]. By using non-purified maturation proteins, the activation reaction volumes could be increased to more than 100 mL, which allowed us to produce and re-purify the milligram quantities of CpI hydrogenase required for spectroscopic analysis.

**Table 1:** Specific activities of CpI activated *in vitro* using natural abundance or isotopically labeled tyrosine.

Tyrosine substrate	CpI (mg·L^−1^)	MV reduction assay†	H_2_ evolution assay†
L-tyrosine	25	683±48	ND*
	50	674±43	ND*
	100	675±79	1649±223
	200	660±45	ND*
L-[1-^13^C]-tyrosine	100	658±50	1840±124
L-[2-^13^C]-tyrosine	100	657±30	1830±392
L-[U-^13^C-^15^N]-tyrosine	100	672±23	1475±375

† CpI activities (µmol H_2_·min^−1^·mg^−1^) were measured using the methyl viologen (MV) reduction assay or the H_2_ evolution assay. The exogenous tyrosine substrates and the CpI apoenzyme concentrations used for *in vitro* [2Fe]_H_ synthesis are provided. Substituting natural abundance L-tyrosine with each isotopically labeled tyrosine analog had no effect on final CpI activities. H_2_ evolution rates, which were measured at the K_m_ for MV (6.25 mM), were nearly half the reported *V*_max_ of 4000 µmol H_2_·min^−1^·mg^−1^ for CpI isolated from *C. pasteurianum* [28]. Also, CpI activities did not decrease when more CpI apoenzyme was added to the reaction mixture. Taken together, these observations indicate nearly complete hydrogenase activation. Data are the average of 3–6 measurements ± standard deviations.

*ND, not determined.

**Table 2:** Summary of energies of the assigned CO and CN^−^ vibrational modes for the CpI hydrogenase H-cluster.

Tyrosine substrate	H-cluster ligand and IR vibrational energy (cm^−1^)				
	(Fe)–CN	(Fe)–CN	(Fe_p_)–CO	(Fe_d_)–CO	μ–CO
L-tyrosine	2082	2070	1970	1947	1801
L-[2-^13^C]-tyrosine	2037 (^13^CN)	2027 (^13^CN)	1968	1947	1801
L-[1-^13^C]-tyrosine	2081	2070	1925 (^13^CO)	1902 (^13^CO)	1761 (μ–^13^CO)
L-[U-^13^C-^15^N]-tyrosine	2006 (^13^C^15^N)	1995 (^13^C^15^N)	1924 (^13^CO)	1902 (^13^CO)	1762 (μ–^13^CO)

**Figure 3:**
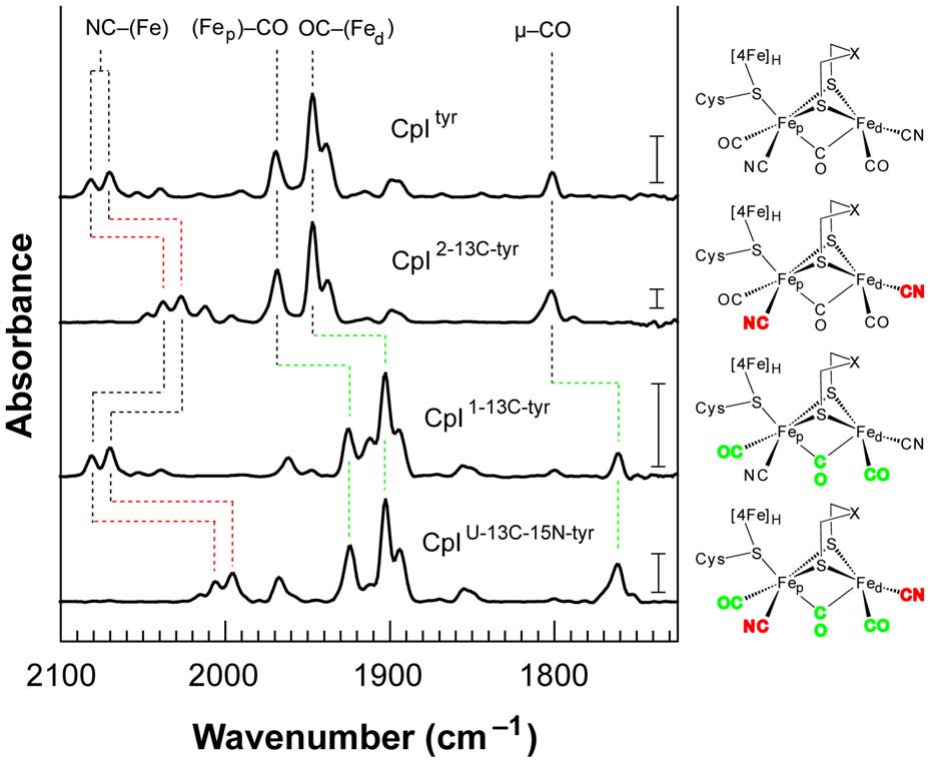
FTIR spectroscopic analysis for active CpI produced *in vitro* using natural abundance tyrosine or isotopically labeled tyrosine analogs. The IR spectra are for the as-isolated active CpI hydrogenase containing the H-cluster produced in the presence of L-tyrosine (CpI^tyr^), L-[2-^13^C]-tyrosine (CpI^2-13C-tyr^), L-[1-^13^C]-tyrosine (CpI^1-13C-tyr^), and L-[U-^13^C-^15^N]-tyrosine (CpI^U-13C-15N-tyr^). The shifts in vibrational energies correlate with expected changes for *ν*(^13^CO), *ν*(^13^CN), and *ν*(^13^C^15^N) modes, confirming that the CO and CN^−^ ligands are synthesized from tyrosine. Labels indicating the assigned *ν*(CO) and *ν*(CN) vibrational modes are provided at the top of the figure, with the ^13^CN^−^/^13^C^15^N^−^ and ^13^CO ligands shown in red and green, respectively, in the molecular diagrams. Vertical scale bars provided at 1740 cm^−1^ represent a difference of 0.5 milliabsorbance units. Table 2 summarizes the vibrational energies and corresponding assigned *ν*(CN) and *ν*(CO) modes for the H_ox_ clusters.

The vibrational energies and corresponding *n*(CN) and *n*(CO) mode assignments are provided for each H_ox_ cluster from active CpI produced with either unlabeled or isotopically labeled tyrosine. Energies were determined from spectra measured using FTIR spectroscopy (Fig. 3). The spectrum for each isotopically labeled sample also contains low intensity bands indicating trace amounts of unlabeled CO and CN^−^incorporated into the H-cluster. The intensities of these bands vary from sample to sample, and they do not depend on the location of either CO or CN^−^ on the H-cluster. We thus attribute these features to either adventitious free tyrosine present in the cell lysates or possibly to low quantities of an iron cluster with CO and CN^−^ ligands that is pre-assembled by a single Hyd maturase during *in vivo* expression. Each spectrum also shows evidence for CpI with reduced H-cluster (H_red_), characterized in the CpI^tyr^ case by bands located at 2053 cm^−1^, 2039 cm^−1^, 1961 cm^−1^, 1914 cm^−1^, and 1899 cm^−1^.

**Table 3:** Summary of energies of the assigned CO and CN^−^ vibrational modes for the CpI hydrogenase H-cluster with bound exogenous CO.

Tyrosine substrate	H-cluster ligand and IR vibrational energy (cm^−1^)				
	(Fe)–CN	(Fe)–CN	(Fe_d_)–CO	(Fe_p_)–CO	μ–CO
L-tyrosine	2090	2076	2015	1973	1807
L-[2-^13^C]-tyrosine	2047 (^13^CN)	2032 (^13^CN)	2012	1972	1806
L-[1-^13^C]-tyrosine	2090	2076	1970 (^13^CO)	1928 (^13^CO)	1767 (μ–^13^CO)
L-[U-^13^C-^15^N]-tyrosine	2015 (^13^C^15^N)	2001 (^13^C^15^N)	1968 (^13^CO)	1928 (^13^CO)	1767 (μ–^13^CO)

**Figure 4:**
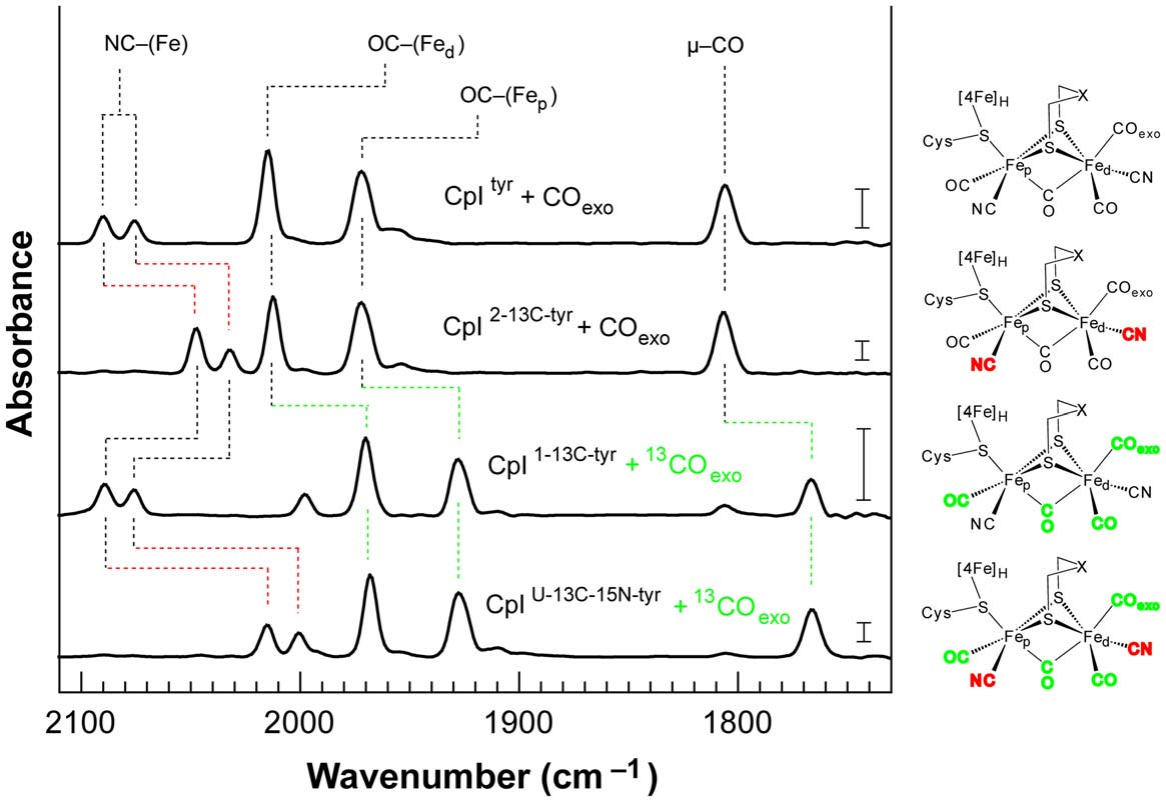
Infrared spectra for CpI hydrogenase with isotopically labeled H-cluster containing exogenously bound CO. The infrared spectra are for the CO-inhibited CpI enzyme harboring an H-cluster produced in the presence of L-tyrosine (CpI^tyr^), L-[2-^13^C]-tyrosine (CpI^2-13C-tyr^), L-[1-^13^C]-tyrosine (CpI^1-13C-tyr^), and L-[U-^13^C-^15^N]-tyrosine (CpI^U-13C-15N-tyr^). Natural abundance CO_exo_ was added to CpI^tyr^ and CpI^2-13C-tyr^, which have intrinsic CO ligands. Conversely, ^13^CO_exo_ was added to CpI^1-13C-tyr^ and CpI^U-13C-15N-tyr^, which have intrinsic ^13^CO ligands. Comparing the H_ox_–CO_exo_ spectrum for each CpI sample to its respective H_ox_ spectrum (Fig. 3), shifts of 5–10 cm^−1^ were observed for the *ν*(CN) modes and the *ν*(μ–CO) mode in all four cases. The *ν*(CO) mode for the Fe_p_–CO ligand did not change. Meanwhile, the *ν*(CO) mode for the Fe_d_–CO moiety was replaced with two peaks resulting from symmetric and asymmetric coupled vibrational stretches, as two CO molecules of equal mass are coordinated to the Fe_d_ atom. The peak for the *ν*(CO)_symmetric_ mode is visible at 2015/1970 cm^−1^ for CO/^13^CO. The *ν*(CO)_asymmetric_ mode, however, cannot be distinguished because its vibrational energy is similar to the *ν*(CO) mode at 1972/1928 cm^−1^ for the Fe_p_–CO/Fe_p_–^13^CO adducts. The changes in vibrational energies, indicated by the dashed lines, correlate with expected changes for *ν*(^13^CO), *ν*(^13^CN), and *ν*(^13^C^15^N) modes, again confirming that the CO and CN^−^ ligands are synthesized from tyrosine. Labels indicating the assigned *ν*(CO) and *ν*(CN) vibrational modes are provided. The ^13^CN/^13^C^15^N and ^13^CO ligands are shown in red and green, respectively, in the molecular diagrams. Vertical scale bars shown at 1740 cm^−1^ represent a difference of 0.5 milliabsorbance units. Table 3 summarizes the vibrational energies and corresponding assigned *ν*(CN) and *ν*(CO) modes for the H_ox_–CO_exo_ clusters.

The vibrational energies and corresponding *ν*(CN) and *ν*(CO) mode assignments are provided for each H_ox_–CO_exo_ cluster from active CpI produced with either natural abundance or isotopically labeled tyrosine. Energies were determined from spectra measured using FTIR spectroscopy (Fig. 4).

Active hydrogenases with either non-labeled or isotopically labeled H-clusters were produced *in vitro* (Table 1) [28], subsequently isolated, and then characterized using FTIR spectroscopy. The coordinated CO and CN^−^ ligands provide well-defined absorption bands that indicate the different chemical states of the H-cluster [29]. Moreover, labeling of CO and CN^−^ with ^13^C and ^15^N alters the observed vibrational energies, providing distinctive fingerprints for tracing which atoms originate from labeled substrates [29], [30].

The IR spectrum of CpI hydrogenase activated *in vitro* with natural abundance tyrosine (Fig. 3, CpI^tyr^) is characteristic for an H-cluster in the oxidized state (H_ox_) [30]. Two peaks at 2082 cm^−1^ and 2070 cm^−1^ derive from the terminal CN^−^ vibrational (*ν*(CN)) stretches. Peaks at 1970 cm^−1^ and 1947 cm^−1^ correspond to the terminal CO (*ν*(CO)) stretches, while the peak at 1801 cm^−1^ indicates the bridging CO (*ν*(μ-CO)) stretch. A nearly identical spectrum has been reported for the CpI hydrogenase isolated from *C. pasteurianum* [30].

IR spectra were next recorded for CpI activated in the presence of tyrosine uniformly labeled with ^13^C and ^15^N isotopes (Fig. 3, CpI^U-13C-15N-tyr^). The peaks for all five *ν*(CO) and *ν*(CN) modes unambiguously shift to lower vibrational energies. Both *ν*(CN) modes decrease by 75–76 cm^−1^ as expected for a two mass unit increase. Both terminal *ν*(CO) modes decrease by 45–46 cm^−1^ as expected for a one mass unit increase. Finally, the bridging *ν*(μ-CO) mode decreases by 39 cm^−1^ also indicating a one mass unit increase. These changes indicate the presence of both the ^13^C and ^15^N isotopes and confirm that all five of the CO and CN^−^ ligands derive from tyrosine.

We then used tyrosine with selectively labeled ^13^C atoms to identify the precise source of the CO and CN^−^ ligands. Reasoning that the CN^−^ ligands originate from the amino group, we produced active CpI using tyrosine labeled only at the amino carbon (Fig. 3, CpI^2-13C-tyr^). The IR spectrum shows that both *ν*(CN) modes decrease by 43–45 cm^−1^, matching the predicted change for terminally coordinated ^13^CN^−^ moieties; all *ν*(CO) modes are unchanged. Therefore, the H-cluster CN^−^ ligands derive from the amino substituent in tyrosine.

Tyrosine contains two carbon atoms with bound oxygen atoms that are plausible sources of the CO ligands: the carboxylic C1 and phenolic C7. CpI was activated in the presence of [1-^13^C]-tyrosine to determine if the CO ligands derive from the carboxylic acid group. The IR spectrum for CpI^1-13C-tyr^ shows that all three *ν*(CO) modes decrease by 40–45 cm^−1^, as previously observed for CpI^U-13C-15N-tyr^, while both *ν*(CN) modes are unchanged. Hence, the IR spectrum for CpI^1-13C-tyr^ clearly illustrates that the H-cluster CO adducts are synthesized from the tyrosine carboxylate substituent.

We also examined the IR spectra for each CpI sample mixed with exogenous CO, which binds to the H-cluster distal Fe atom. The CO binding causes well-characterized changes in the spectrum [29], [30], and the shifts in the *ν*(CO) and the *ν*(CN) modes that we observed support our previous assignments and interpretations (Fig. 4).

Reconstituting the H-cluster biosynthetic pathway using a *Clostridial* hydrogenase, *Shewanella* maturases, and *E. coli* lysates highlights the modularity of the hydrogenase maturation system and suggests that the mechanisms for CO and CN^−^ ligand synthesis for [FeFe] hydrogenases may be broadly conserved. Questions still remain, however, as to how CO and CN^−^ are synthesized from tyrosine and subsequently coordinate to an iron cluster. The formation of a radical at the tyrosine C7 hydroxyl group could lead to either a glycyl radical or a reactive dehydroglycine intermediate [31], and such radical SAM chemistry has precedence given the requirement for the para-hydroxyl substituent of tyrosine for *in vitro* H-cluster synthesis [23]. Recent investigations comparing the wild-type and a mutant HydG maturase have provided further insights into the mechanism for CO and CN^−^ synthesis, and the authors proposed that a glycyl radical is the more likely intermediate derived from tyrosine [17].

As we have shown, reconstituting biosynthetic pathways using cell lysates can lead to new insights, yet establishing *in vitro* systems containing purified enzymes and a defined set of substrates can also be important for understanding biochemical conversions [20]. Interestingly, the hydrogenase maturation pathway could not be reconstituted when using purified HydE–*Strep-*tag II, HydF–*Strep*-tag II, and *Strep*-tag II–HydG combined with Fe^+2^, S^−2^, SAM, cysteine, tyrosine, DTT, GTP, PLP, and dithionite. An *E. coli* cell lysate without any maturases was also required with these constituents to activate *in vitro* H-cluster synthesis and hydrogenase maturation. This difference indicates that uncharacterized components of the *E. coli* lysates are necessary, perhaps proteins involved in Fe–S cluster synthesis.

The roles of the small molecule substrates also require further investigation. Compared to our previous system, four additional chemicals were beneficial for high-yield CpI activation. These include two reducing agents (DTT and sodium dithionite), GTP, and PLP. Dithionite is likely an electron source for the maturase-based radical SAM chemistry [16], [18], [32], [33]. A GTP requirement is also not unexpected as HydF is a GTPase, although high concentrations of this nucleotide (>10 mM) were needed when maturing micromolar concentrations of the [FeFe] hydrogenases. We also observed that GTP could be replaced by ATP, though nucleoside diphosphate kinase activity from the *E. coli* lysates might be regenerating GTP from GMP and GDP. The third substrate, PLP, may be a cofactor of the maturases, although it is more likely contributing as a cofactor for cysteine desulfurases such as NifS and IscS, which may be facilitating cell-free Fe–S cluster synthesis [34]. This interpretation is supported by the observation that cysteine also enhances *in vitro* hydrogenase activation [23].

The *in vitro* system we have described can also be used for studying the maturases. For example, we replaced the HydF lysate with one containing an affinity-tagged maturase (HydF–*Strep*-tag II). Following cell-free H-cluster synthesis in the absence of the CpI hydrogenase, we purified the HydF protein to greater than 95% purity and hypothesized that it could have a bound H-cluster precursor [13], [25], [35]. Interestingly, the purified HydF showed hydrogenase-like activity, with the ability to evolve hydrogen (1.5 µmol H_2_ produced·min^−1^·mg^−1^ HydF) as well as to reduce methyl viologen in the presence of 2% H_2_ (1.2 µmol MV reduced·min^−1^·mg^−1^ HydF, likely by H_2_ uptake). The catalytic rates are less than 1% of those from the active CpI hydrogenase, but identical reaction mixtures lacking both HydF and the CpI apoenzyme showed no detectable activity. Therefore, the HydF activities indicate that this maturase contained an *in vitro* synthesized H-cluster precursor.

This report provides the first example of cell-free H-cluster synthesis and hydrogenase activation using individually expressed maturases, and it also clearly details the origin of all five H-cluster CO and CN^−^ ligands. Furthermore, our results underscore the utility of this *in vitro* approach for follow-up studies such as ^57^Fe labeling for Mossbauer spectroscopy as well as attempts to determine the origin of the H-cluster dithiolate ligand. One hypothesis is that the bridge also derives from tyrosine [32], and we are now in a position to directly examine this possibility.

## Materials and Methods

### Materials and Chemical Solutions

Isotopically labeled L-[1-^13^C]-tyrosine, L-[2-^13^C]-tyrosine, and L-[U-^13^C-^15^N]-tyrosine were obtained from Cambridge Isotope Laboratories, Inc. Fresh solutions of SAM, L-tyrosine, L-cysteine, GTP, sodium dithionite, and PLP were routinely prepared with anaerobic buffers before all *in vitro* studies. SAM was dissolved in 10% ethanol and 5 mM sulfuric acid. All other additives were dissolved in 50 mM Hepes buffer, and the final pH was adjusted to 7.0–8.0.

### Expression Constructs

*S. oneidensis* maturase genes *hydE*, *hydF*, and *hydG* were PCR amplified from the pACYCDuet-1–*hydGX*–*hydEF* construct [23], and the [FeFe] hydrogenase gene *hydA* from *C. pasteurianum*, previously codon-optimized for expression in *E. coli*, was amplified from the pK7 *shydA* vector [36]. All PCR products were subsequently cloned into the pACYC plasmid (Novagen) or the pET-21(b) plasmid (Novagen), and the following constructs were made: pACYC *hydE*, pET-21(b) *hydE–Strep*-tag II, pET-21(b) *hydF*, pET-21(b) *hydF–Strep*-tag II, pACYC *hydG*, pET-21(b) *Strep*-tag II–*hydG*, pET-21(b) *hydG–Strep*-tag II, pET-21(b) *Strep*-tag II*–shydA*, and pET-21(b) *shydA–Strep*-tag II. Proteins containing an N-terminal or C-terminal *Strep*-tag II® affinity tag (IBA GmbH) with a two residue linker have the added peptide sequence 5′-WSHPQFEKSA-3′ or 5′-SAWSHPQFEK-3′, respectively. All expression constructs were individually transformed into *E. coli* strain BL21(DE3) Δ*iscR::kan*. The engineered Δ*iscR* strain has been shown to improve recombinant expression of Fe–S proteins [37], and more recently to produce high yields of active [FeFe] hydrogenases [26].

Expression of *Strep*-tag II–CpI did not result in the production of soluble full-length hydrogenase. The maturase HydG–*Strep*-tag II expressed as a soluble protein, but did not function with HydE and HydF to activate the [FeFe] hydrogenase *in vitro*. Prior to this work, the HydF*–Strep*-tag II maturase was expressed in *E. coli* from the plasmid pACYCDuet-1–*hydGX*–*hydEF–Strep-tag II*, and then purified. Edman degradation of HydF–*Strep*-tag II revealed an N-terminal sequence and translation start site different than previously suggested (Accession # AAN56901). The protein sequence of the HydF maturase used in this work is provided in the supporting information as Figure S1 (also Accession # ADK73963). Sequences for the HydE, HydF, and HydG maturases have been deposited in the National Center for Biotechnology Information GenBank (accession codes HM357715, HM357716, and HM357717).

### Maturase Lysate and Hydrogenase Apoenzyme Preparations

Batch fermentations were performed using a 5 L BioFlo 3000 fermentor (New Brunswick Scientific) as described previously [26]. 4 L of LB Miller complex growth medium also contained 50 mM MOPS buffer, 25 mM glucose, 500 mg·L^−1^ ferric ammonium citrate, and the appropriate antibiotics (pH 7.4). Cells were aerobically grown (25°C, 4 SLPM airflow) until the OD_600_ reached 0.5–0.7. At this time, gas flow was changed to 100% N_2_ at 2 SLPM, agitation speed was changed from 500 to 100 rpm, and both 10 mM sodium fumarate and 2 mM L-cysteine were added to the culture. After 15 min, strict anoxic expression of heterologous protein was induced with 0.5 mM IPTG for 12 hr. The final OD_600_ of cultures ranged from 1.6 to 2.4.

Following maturase or hydrogenase apoenzyme expression, cells were harvested, pelleted, and lysed while maintaining anaerobic conditions. An anaerobic glove box (Coy Laboratory Products) containing 98% N_2_ and 2% H_2_, generally at 25–27°C, was used for all *in vitro* work. Cells were resuspended in BugBuster® Master Mix lysis solution (4 mL per gram of wet-cell paste) supplemented with 50 mM Hepes buffer (pH 8.2), 50 mM KCl, 2 mM dithionite, and 2 µM resazurin. After 30 min, lysates were clarified at 20,000×g. Maturase lysates (HydE^lysate^, HydF^lysate^, and HydG^lysate^) were sealed anaerobically, flash frozen using liquid N_2_, and stored at −80°C.

Purification of the CpI apoenzyme and the maturases was done following lysate clarification using *Strep*-Tactin® Superflow® high capacity resin (IBA GmbH) equilibrated with 50 mM Hepes buffer (pH 7.8) and 100 mM KCl. CpI yields after purification were 10–20 mg·L^−1^ culture, and apoenzyme solutions were concentrated to 3–6 mg·mL^−1^ (50–100 µM) using a stirred cell concentrator and a 5 kD membrane (Amicon). Concentrated apoenzyme was subsequently buffer exchanged using PD-10 desalting columns (GE Healthcare) to remove the D-desthiobiotin. Solutions of purified proteins were sealed anaerobically, flash frozen using liquid N_2_, and stored at −80°C.

### *In Vitro* Activation of Active [FeFe] Hydrogenases

Anaerobic reaction mixtures varied from 50 µL to 100 mL, depending on the experiment, and hydrogenase activation proceeded over a 24 hr period. Equivalent CpI activities were observed within this range of volumes. The reaction mixtures included HydE^lysate^, HydF^lysate^, HydG^lysate^, exogenous substrates, and CpI apoenzyme. Fe^+2^, S^−2^, and DTT were first added to the mixture of maturase lysates. After 30 min, additional small molecule substrates and CpI apoprotein were added. The final concentration for each component was as follows: 20% vol·vol^−1^ HydE^lysate^, 20% vol·vol^−1^ HydF^lysate^, 20% vol·vol^−1^ HydG^lysate^, 1 mM Fe^+2^, 1 mM S^−2^, 1 mM DTT, 2 mM SAM, 2 mM L-cysteine, 2 mM tyrosine, 10 mM GTP, 1 mM PLP, 2 mM sodium dithionite, and 0.2 mg·mL^−1^ CpI apoenzyme. We estimated the *E. coli* lysates to have 3–15 mg·mL^−1^ of each maturase based on SDS-PAGE analysis (Fig. 2). Therefore, *in vitro* reaction mixtures contained ∼10–50 µM of HydE (40 kD), HydF (45 kD), and HydG (54 kD). The purification and concentration of active CpI holoenzyme was carried out as described above for CpI apoenzyme. Solutions of 100–300 µM active CpI were analyzed with FTIR spectroscopy.

### Hydrogenase Activity Assays

Both the H_2_ consumption and H_2_ evolution rates for activated hydrogenase were measured as previously described [23], [36], with or without re-purifying the active CpI. H_2_ uptake rates were measured with a methyl viologen (MV) reduction assay and calculated using an extinction coefficient of 9.78 mM^−1^·cm^−1^ for reduced MV at 578 nm. The assay solution contained 50 mM Tris/HCl (pH 8.0) and 2 mM MV. The H_2_ evolution assay solution included 100 mM MOPS buffer, 100 mM NaCl, 25 mM sodium dithionite, and 6.25 mM MV. H_2_ production rates at pH 6.8 and 37°C were quantified by analyzing head space gas samples using a ShinCarbon ST 100/120 mesh column (Resteck) with a Hewlett Packard 6890 gas chromatograph (Hewlett Packard). For precise activity measurements, approximately 1 ng and 10 ng of CpI were tested with the MV reduction and H_2_ evolution assays, respectively. Background activities (less than 1% of the final activity from mixtures will all components) were measured for mixtures containing all components except the hydrogenase, and the CpI apoenzyme had neither H_2_ production nor H_2_ oxidation activity.

### Fourier Transform Infrared Spectroscopy

Infrared spectra were measured using a Bruker IFS/66s FTIR spectrometer interfaced to a home-built stopped-flow drive system as previously described [38]. The drive system and infrared sample cuvette were maintained inside an anaerobic glove box (O_2_<1.1 ppm) (Belle Technology) at 25°C. A calibrated path length of 47.6 µm was used for the sample cuvette. For infrared spectroscopic measurements, one drive syringe contained the protein sample. Depending on the experiment, the second drive syringe contained one of the following: the same protein sample, the purification elution buffer without protein, elution buffer saturated with exogenous ^12^CO, or elution buffer saturated with exogenous ^13^CO. Spectra were recorded at 4 cm^−1^ resolution, and an arbitrary background correction was applied. The IR data were processed and analyzed using the Fit_3D software package (SJG, unpublished).

## Supporting Information

Figure S1 ***Shewanella oneidensis*** **HydF protein sequence based on recombinant expression of the** ***S. oneidensis hydEF*** **open reading frame in** ***Escherichia coli*** **.** The underlined peptide sequence corresponds to the residues added to the N-terminus of the previously published *S. oneidensis* HydF peptide sequence (Accession # AAN56901). The amino acids highlighted in black bold font type correspond to the residues identified by Edman degradation and N-terminal sequencing of HydF–*Strep*-tag II when expressed in *E. coli* strain BL21(DE3) from the plasmid pACYCDuet-1–*hydGX*–*hydEF–Strep-tag II*. The consensus sequences for the GTP binding motif are depicted in green bold font type, which now appear more accurately aligned with sequences of HydF maturases from other organisms [39].
(TIF)
