# Supplementary figures and images for: Cell-free H-cluster Synthesis and [FeFe] Hydrogenase Activation: All Five CO and CN− Ligands Derive from Tyrosine
Source: PLoS One. 2011 May 31;6(5):e20346. doi: 10.1371/journal.pone.0020346 (PMC3105041; doi:10.1371/journal.pone.0020346)

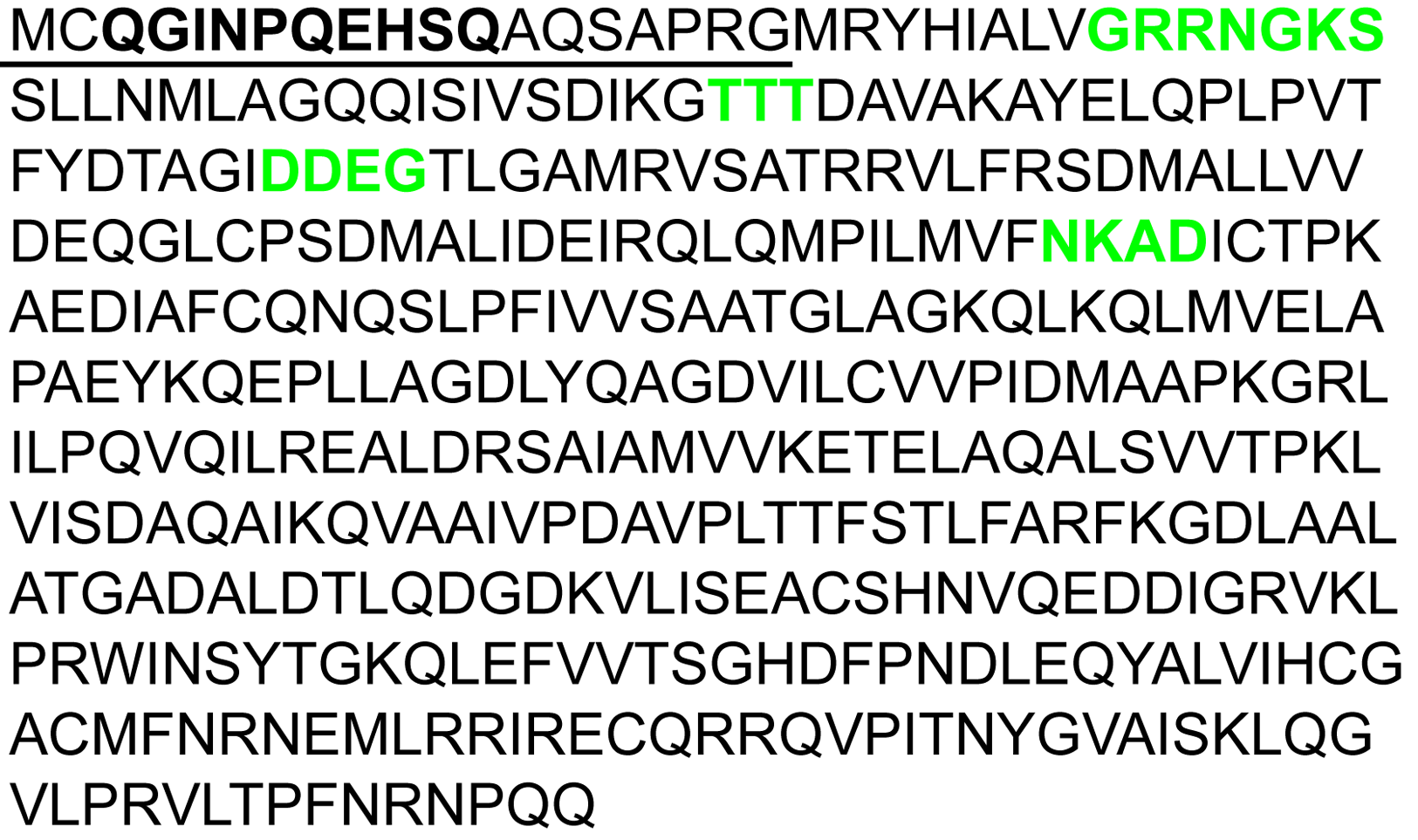

Supplement: Figure S1 — Shewanella oneidensis HydF protein sequence based on recombinant expression of the S. oneidensis hydEF open reading frame in Escherichia coli . The underlined peptide sequence corresponds to the residues added to the N-terminus of the previously published S. oneidensis HydF peptide sequence (Accession # AAN56901). The amino acids highlighted in black bold font type correspond to the residues identified by Edman degradation and N-terminal sequencing of HydF–Strep-tag II when expressed in E. coli strain BL21(DE3) from the plasmid pACYCDuet-1–hydGX–hydEF–Strep-tag II. The consensus sequences for the GTP binding motif are depicted in green bold font type, which now appear more accurately aligned with sequences of HydF maturases from other organisms [39]. (TIF) [file pone.0020346.s001.tif]
